# Glass Fiber Reinforced Polypropylene Mechanical Properties Enhancement by Adhesion Improvement

**DOI:** 10.3390/ma5061084

**Published:** 2012-06-18

**Authors:** Mariana Etcheverry, Silvia E. Barbosa

**Affiliations:** Planta Piloto de Ingeniería Química, PLAPIQUI (UNS-CONICET), Cno. La Carrindanga Km. 7, Bahía Blanca 8000, Argentina

**Keywords:** glass fiber/polypropylene composites, *in-situ* polymerization, improvement adhesion, mechanical properties

## Abstract

Glass fibers (GF) are the reinforcement agent most used in polypropylene (PP) based composites, as they have good balance between properties and costs. However, their final properties are mainly determined by the strength and stability of the polymer-fiber interphase. Fibers do not act as an effective reinforcing material when the adhesion is weak. Also, the adhesion between phases can be easily degraded in aggressive environmental conditions such as high temperatures and/or elevated moisture, and by the stress fields to which the material may be exposed. Many efforts have been done to improve polymer-glass fiber adhesion by compatibility enhancement. The most used techniques include modifications in glass surface, polymer matrix and/or both. However, the results obtained do not show a good costs/properties improvement relationship. The aim of this work is to perform an accurate analysis regarding methods for GF/PP adhesion improvement and to propose a new route based on PP *in-situ* polymerization onto fibers. This route involves the modification of fibers with an aluminum alkyl and hydroxy-α-olefin and from there to enable the growth of the PP chains using direct metallocenic copolymerization. The adhesion improvements were further proved by fragmentation test, as well as by mechanical properties measurements. The strength and toughness increases three times and the interfacial strength duplicates in PP/GF composites prepared with *in-situ* polymerized fibers.

## 1. Introduction

Glass fibers (GF) are the most common reinforcement for polymeric matrix composites. Their principal advantages are the relationship between their low cost, high tensile strength, high chemical resistance, and insulating properties. The disadvantages are low tensile modulus, relatively high specific gravity, sensitivity to abrasion during handling, low fatigue resistance, and high hardness. E-glass and S-glass are the types of fibers more commonly used in the fiber-reinforced plastic industry. E-glass fibers have the lowest cost of all commercially available reinforcing GFs, which is the reason for their widespread use in the fiber-reinforced plastic industry. S-glass, originally developed for aircraft components and missile casings, has the highest tensile strength among all fibers in use. However, the compositional difference and higher manufacturing cost make it more expensive than E-glass [1,2,3].

The manufacturing process for GFs includes several steps. Various ingredients in the glass formulation are first dry mixed and melted in a refractory furnace at about 1,370 °C. The molten glass is exuded through a number of orifices contained in a platinum bushing and rapidly drawn into filaments of approximately 10 µm diameter. A protective coating (“size”) is then applied on individual filaments before they are gathered together into strands and wound on a drum. The size is a mixture of lubricants, which prevent abrasion between the filaments, antistatic agents, which reduce static friction between the filaments and a binder, which packs the filaments together into a strand. It may also contain small percentages of a coupling agent that promotes adhesion between fibers and the specific matrix for which it is formulated [1].

Fiber-reinforced composite materials consist of fibers with high strength and modulus embedded in a matrix. Both fibers and matrix retain their physical and chemical identities and the new material has a combination of properties that cannot be achieved with either of the constituents acting alone. In general, fibers are the main load-carrying members, while the matrix functions are: to transfer stresses between the fibers, to provide a barrier against an adverse environment, and to protect the fiber surface from mechanical abrasion [1,3].

Polymer composites have been used in a wide range of industrial applications like aeronautic, naval, construction, sporting goods, home appliances, furniture, *etc.* They have been on the market for over fifty years, and have generally been used to replace materials such as wood, aluminum and steel. Their advantages over traditional materials include greater mechanical strength, lighter weight, better dimensional stability, higher dielectric strength and corrosion resistance, and flexibility to improve the designs [4]. The major advantage of these composites that helped them enter into such a variety of markets is their high specific property, which is greater than that of metals and ceramics. Nevertheless, the tailoring of well-bonded and/or durable interphases between the matrix and reinforcement remains a critical issue in this kind of material. This factor is critical with thermoplastic polymer matrices such as polyethylene (PE), polypropylene (PP), polyvinylchloride (PVC), polystyrene (PS) and polyamide (PA).

The effectiveness of reinforcement essentially depends on the adhesion between matrix and fiber, so this is a key factor in determining the final properties of the composite material, particularly its mechanical properties [1,5,6,7]. The fiber-matrix adhesion is confined to a region or “third phase” known as interphase, where stress-transfer occurs. The interphase is defined as the tridimensional region, located between the fiber and the matrix. It is considered as a transition region or third phase with its own characteristics, corresponding neither to fiber properties nor to matrix ones [8]. Wu postulated that the extent of molecular or local segmental diffusion across the interface determines the structure of the interfacial zone, which critically affects the mechanical strength of an adhesive bond. Negligible diffusion will give a sharp interface with weak adhesion. In this case, high adhesive strength can be expected only when strong polar interactions or chemical bonds exist across it. On the other hand, if the interphase is relatively thick and gradual as a result of extensive diffusion, a high adhesive strength will result just by the effect of dispersion forces [9].

## 2. Methods for Adhesion Improvement

The strengthening of matrix-reinforcement interphases has been the goal of a great amount of research, particularly in thermoplastic composite materials. Different kinds of treatments are necessary to improve the interfacial adhesion [10]. Compatibility can be increased by fiber surface treatment, changes in the polymer matrix or both [1,11]. Among the compatibilizing methodologies, the most widely used is fiber treatment with coupling agents and techniques for modifying the matrix, such as alkali treatment, acetylation and graft copolymerization, which are summarized below [12].

### 2.1. Glass Fiber Treatment

The glass surface modifications by treatment with a coupling agent are used to improve the fiber/matrix interfacial strength through both physical and chemical bonds, and to protect the fiber surface from environmental conditions, such as moisture and reactive fluids. Organofunctional silanes are the most widely used coupling agents for improvement of the interfacial adhesion in glass-reinforced materials. Their effectiveness depends on the nature and pretreatment of the substrate, the silane type, the silane layer thickness and the application process. In a relatively dry state, the proper selection of a silane coupling agent is an effective means of promoting interfacial adhesion and enhancing mechanical properties. Under wet conditions, however, its effectiveness substantially depends on the nature of the chemical bond between the silane coupling agent and the primary constituents, *i.e.*, the GFs and the polymeric matrix. A variety of mechanisms have been proposed to explain the function of silanes at the interphase. Plueddemann and colleagues were the first to perform a systematic study of the effectiveness of over a hundred silane coupling agents on the wet strength of epoxy and polyester laminates [13,14,15,16]. These data suggest that the primary factors are:

- The chemical reactivity of the silane organofunctional group to form covalent bonds with the polymer matrix.
- The primary or secondary chemical bond formation at the glass interface.
- The ability of the polymer matrix to diffuse into the silane “interphase” to form a rigid, tough, water resistant interpenetrating polymer network as a transition zone between the bulk matrix and glass reinforcement.

The coupling mechanism of these agents has not been fully clarified, probably because of the complex nature of the interfacial interactions [1,5,7,11]. The chemical bonding theory states that silane undergoes a chemical reaction with the surface of inorganic substances to form Si-O-M bonds (where M is either a Si atom from glass or metallic atom in general). This theory was verified for silanes reacting with silica, where the alkoxysilanes undoubtedly formed Si-O-M bonds. Since glass consists mainly of SiO_2_, silanol and siloxane groups, they are likely to be present at the GF surface. When heated, silanol groups decompose into siloxano by releasing water. Also siloxane groups, formed at moderate temperatures, will rehydrate in the presence of water, to form silanol groups. In any case, the conditions of glass surfaces depend on the environment, particularly on the moisture content. It can be expected that a few layers of free water (thickness in the range of the diameter of the water molecule) remain weakly adsorbed. In unsaturated polyesters-silane-treated GF composites, the interphase strength depends on the reactivity of the agents to the unsaturated polyesters. This fact strongly suggests the formation of chemical bonds between the resin and the coupling agents. Instead, the last ones have less effect on thermoplastic resins, in particular on polyolefins, for which there are no specific reactions that may involve silanes. Studies on silane coatings include the development of aminosilane with an unsaturated bond, carboxylic acid functional silane, cationic silane, silyl peroxide, and aminimide.

Zulkifli investigated the effect of different types of silane and their concentrations in the interphase chemistry of the filler particle or fiber reinforcement in a polymer matrix and their influences on the fracture properties. Surface fracture analysis was carried out to determine the level of fiber-matrix debonding. It was found that physicsorbed silane removing enhances its chemisortion in order to form bonds with the substrate and improve the fiber-matrix adhesion [17].

Other coupling agents used with GFs are titanium compounds, known as titanates. They are mainly used to modify the surface of mineral reinforcements such as calcium carbonate, talc, *etc*. in order to make them compatible with resins such as PP, PE, PS, and to use them as processing aids [1,5,16,18,19]. These coupling agents are more effective than silanes with thermoplastic resins because they have three functional groups compared to only one in the silanes. In addition, titanates also act as plasticizers to allow higher rates of charges and/or to achieve a better flow. When applied as a coating, they reduce the viscosity of the system and increase the reinforcement concentration. Their main disadvantage is the very high cost, which is much higher than that of silanes.

### 2.2. Matrix Modifications

Another way to improve the thermoplastic/glass fiber interphase is to add a specific modified polymer to the matrix. Taking into account that polyolefins are highly non-polar, the introduction of polar functional groups to the PP chains can improve compatibility with polar strengthening and achieve a homogenous dispersion of additives, fillers and reinforcements. The polar groups in PP chains are introduced by reactions with species that contain specific functional groups in their structures, such as ester, carboxylic acid or anhydride groups. This kind of strategy is used for PP/GF composites in which a maleic anhydride grafted polypropylene (PP-g-MA) is added in order to react with the amine group of the silanized glass surface [20,21]. A similar methodology was adopted by Di Benedetto *et al.* who used oligomers of poly(sulfone) and poly(carbonate) to promote the fiber bonding with different matrices. In these cases, the coupling proceeds from the reaction between organic functions of the silanized glass surface and the modified oligomer [22]. Lee *et al.* [23] evaluated the ability of hydroxylated polypropylenes to improve the adhesion between pure PP and glass laminates. They found evidence for chemical bonding between PP-OH and glass surfaces with a subsequent interfacial interaction. Van den Oevert and Peijs investigated the influence of PP-g-MA on fatigue behavior in continuous glass-fiber-reinforced polypropylene (GFRPP) composites. In the case of longitudinal tension-tension fatigue, only in a slightly higher absolute fatigue strength results of the use of PP-g-MA. In the case of off-axis (shear or transverse) fatigue, improved adhesion as a result of the use of PP-g-MA results in significantly higher fatigue strength [24].

Rijsdijk *et al.* [20] investigated the influence of PP-g-MA on monotonic mechanical properties in continuous GFRPP. Three-point bending tests were performed on 0° and 90° unidirectional glass-fiber/PP laminates with various weight fractions of PP-g-MA in the PP matrix. These tests showed an increase in both longitudinal and transverse flexural strength up to 10 wt% PP-g-MA, whereas at higher PP-g-MA weight fractions, a flexural strength decrease was observed. No significant influence of PP-g-MA on composite stiffness was found. Additional mechanical tests on unidirectional glass/PP composites with 0 wt% and 10 wt% PP-g-MA showed only a small increase in fiber-dominated properties such as longitudinal tensile strength and strain, whereas composite properties that are governed by the interphase, such as transverse, shear and compressive strength, showed significant increases as a result of matrix modification and an enhanced interaction between the GFs and the PP matrix.

Bogoeva-Gaceva *et al.* also investigated the characterization of a PP-g-MA as an adhesion promoter for GF composites. They found that the apparent shear strength of single-fiber model composites with PP-g-MA-modified iPP was increased compared with those with homo-iPP [25].

It is important to note that, during PP-g-MA preparation, melted PP is subject to a grafting reaction with maleic anhydride in the presence of peroxide initiators. The use of peroxide initiators together with the required processing conditions causes the formation of products not completely homogeneous and characterized by a PP degradation, which makes them unsuitable to use as a matrix or, due to the not guaranteed constant and repeatable properties, as compatibilizing agents in the preparation of GFRPP. This fact makes PP-g-MA only attractive as an additive to blend with pure PP [26].

### 2.3. Fiber Treatment and Matrix Modifications

Several studies have been performed in order to determine the influence of the interphase PP/GF on the mechanical properties by modifying the fibers with γ-aminopropyltriethoxysilane and various polymer dispersions (PE/polyurethane), PP, PP/polyurethane and epoxy) with PP-g-MA. The experimental results showed that physical and chemical interactions established in the composite improved the interfacial strength values and mechanical properties in all cases [21,27,28,29,30,31,32,33]. Feller *et al*. [34,35] investigated a way to increase mechanical properties of PP/GF composites modifying the fiber/matrix interphase using model multifunctional sizing agents (triethoxy and chlorodimethyl silane grafted isotactic copolymers of propene and dienes). These polymers were synthesized through heterogeneous Ziegler-Natta catalysis copolymerization and hydrosilylation with a Speier catalyst. The resulting multifunctional sizing agents can create chemical bonds with the fiber surface and co-crystallize with the PP matrix (co-crystallization is obtained between polymers having very similar chemical and crystalline structures). They found that, for ramificated poly(propene)s it is possible to adjust the surface free energy with the silane content. The more important interfacial shear stress was obtained with poly(propene-co-8.5%methyloctadiene-g-chlorodimethylsilane) showing that a compromise must be found between the silane content and polymer crystallinity. The main disadvantage of these methods is the relationship between high-costs and low efficiency.

## 3. Fiber *In-Situ* Polymerization as a Route of Adhesion Improvement

PP polarity is so low then the adhesion with GF is bad. As it was shown above, a lot of effort was done in order to enhance the compatibility between both surfaces, but improvement obtained was low. Also its viscosity in the molten state is so high that good penetrability in GF mats is impeded. The best possible adhesion is given by chemical anchorage between the polymer and fiber. Following this idea, PP was polymerized and chemically bonded on the glass surface [36,37] in our group. This reaction involves a metallocenic catalysis starting with propylene gas. In this sense, when polymerization is performed onto the glass-fiber mat, the penetrability of this gas is so high that PP “prepreg” mats could be obtained [38].

**Figure 1:**
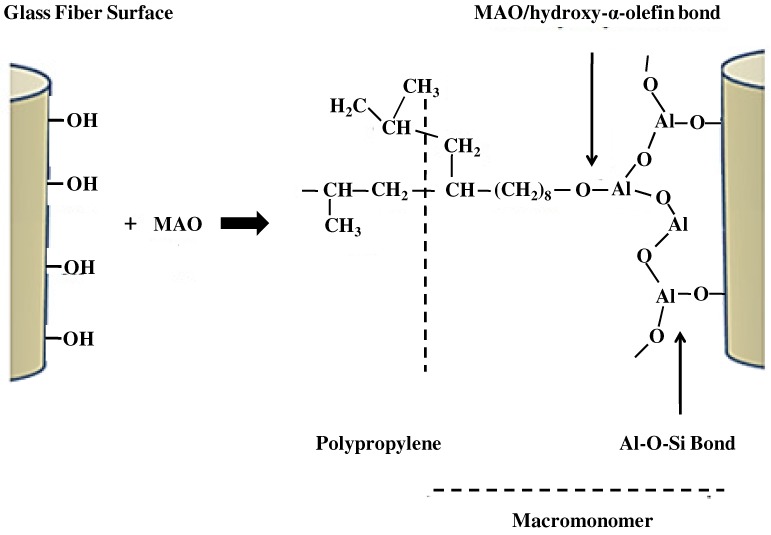
Scheme proposed for the polypropylene (PP) grafting molecules onto glass surface.

The experimental reaction route involves an initial contact with methylaluminoxane (MAO) and hydroxy-α-olefin (9-decen-1-ol) to generate the anchorage points on the fiber surface, followed by a propylene polymerization catalyzed by EtInd_2_ZrCl_2_ (metallocene)/MAO. The reaction was carried out onto GFs of about 25 μm nominal diameter, provided by Vetrotex Argentina. It should be noted that, because the extruding glass process takes place at very high temperatures on the surface, hydrolysis used for all surface modifications, silanization, titanization, and so on, and will also be used as anchor points for the proposed direct copolymerization in this study. Figure 1 shows the reaction scheme proposed for the PP grafting molecules onto glass surface.

The graft reaction of PP onto GFs includes two steps:

- *Glass-MAO pretreatment*. The first step is MAO addition in order to produce an appropriate concentration of CH_3_ groups (from the MAO), which subsequently react with the compounds to be copolymerized (the hydroxy-α-olefin) through the OH groups. The glass reacts with MAO through the OH groups anchored on its surface, releasing methane (CH_4_) and generating a bond Al-O-Si (glass). The methyl group on the (glass) Si-O-Al-CH_3_ reacts with the OH of the hydroxy-α-olefin, releasing CH_4_. The final anchorage on the surface, previous to the propylene polymerization reaction, is AlO_3_, with one of the O of the AlO_3_ linked to an alkyl group and the other two to Si or Al. The addition of a hydroxy-α-olefin to supply the necessary vinyl-bonds to initiate the propylene polymerization is performed. It was demonstrated that short hydroxy-α-olefins (5-hexen-1-ol) [39] tend to act as Lewis bases; however the use of long-chain hydroxy-α-olefins (9-decen-1-ol,10-undecen-1-ol) is preferred to assure a low level of poisoning [40].
- *Reaction of the glass surface with hydroxy-α-olefin*. After the MAO reaction to the glass surface, hydroxy-α-olefin is added. The hydroxy-α-olefin reacts with the MAO-CH_3_ groups with evolution of methane and the setting of terminal vinyl groups to the glass. Then, a copolymerization reaction can be initiated from these macrocomonomers. The propylene (CH_3_–CH=CH_2_) molecules are attached via catalytic polymerization using EtInd_2_ZrCl_2_ (metallocene)/MAO. The MAO is included in the metallocenic catalyst to alkylate the metallocene, and to generate and stabilize the cationic active zirconocene. As a result, PP chains grow by copolymerization of propylene with the vinyl group of the Al-O(CH_2_)n-C=CH_2_, remaining chemically bonded to the glass. Finally, the polymer precipitates by acidified ethanol aggregate. Details of the experimental procedure are described in previous works [36,38]. This reaction scheme (Figure 1) was further proved by consistent evidence obtained by combining scanning electron microscopy techniques (SEM) with X-ray energy dispersive analysis (EDX).

**Figure 2:**
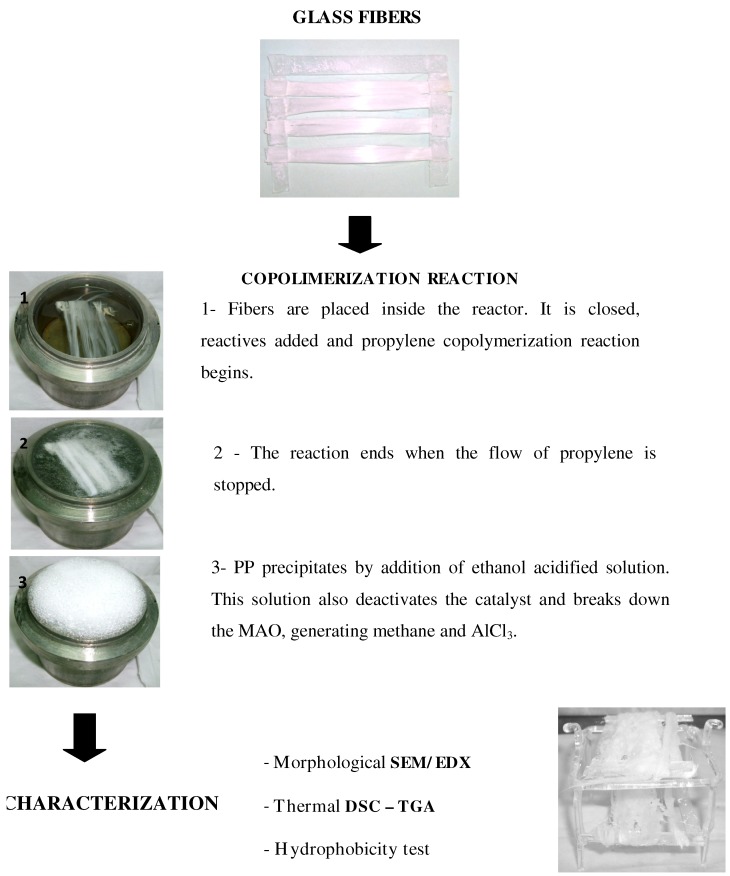
Experimental methodology.

The hydroxy-α-olefin concentration used in each case was calculated in order to ensure the activity of the metallocene catalyst. The metallocene concentration remained constant. Therefore, the reaction variables were selected aimed to obtain a high level of PP coverage on the glass surface by chemical bonding between them. Table 1 summarizes the nomenclature of all reactions performed, where the concentrations in percentage were calculated following the calculations explained in reference [37]. The experimental methodology, as well as the techniques used for characterization, are schematically described in Figure 2.

**Table 1:** Nomenclature of all reactions performed.

Name	Hydroxy-α-olefin [μL]	Total MAO [mL]
F0	----	----
F0.5%	50	10
F1%	100	10
F1.5%	150	12
F2%	200	12

In order to determine the reaction occurrence, a comparative study was performed on fiber surfaces after strong PP extraction. In a previous work, screening experiments were performed using extremely low and high hydroxy-α-olefin concentrations, 0.4 and 4%, respectively. In these studies, it was demonstrated that propylene effectively copolymerizes onto fiber surfaces and the final morphology depends on the hydroxy-α-olefin amount used during the reaction [36,41].

## 4. Morphological Aspects of *in Situ* Polymerized Fiber Glass

**Figure 3:**
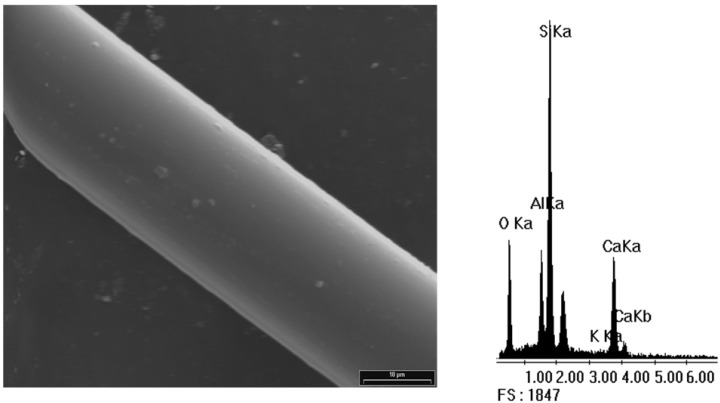
Scanning electron micrograph (SEM) of the sample F0 (2,000×) with its corresponding EDX spectrum.

**Figure 4:**
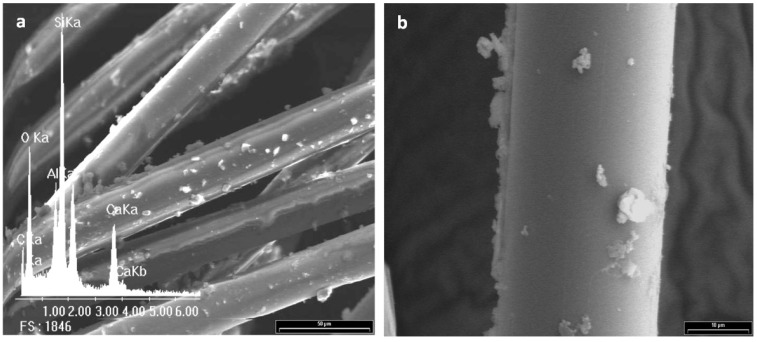
SEM micrographs for sample F0.5% and its corresponding EDX spectrum. (**a**) 600× and (**b**) 2,000×.

**Figure 5:**
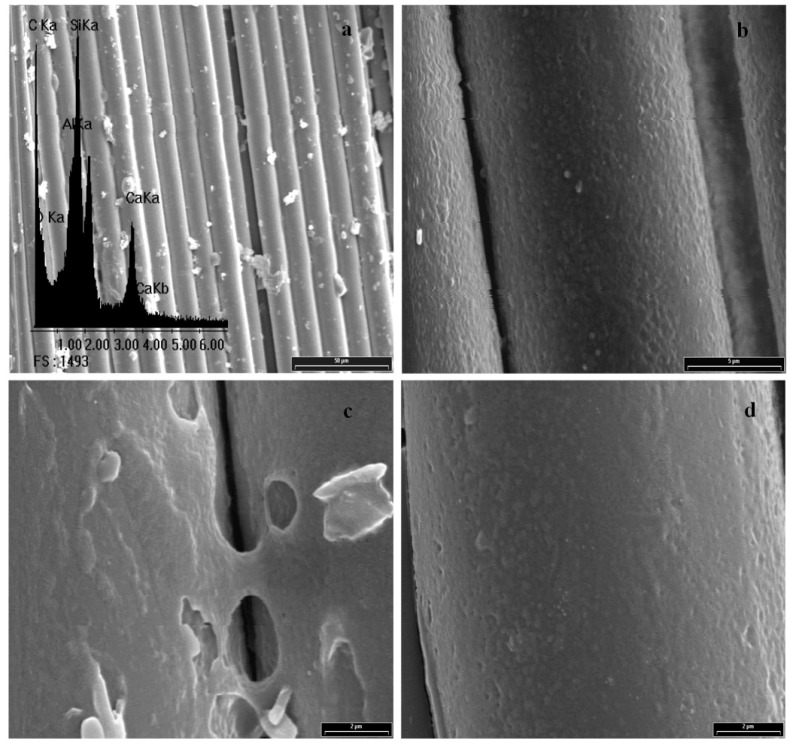
SEM micrographs for sample F1% and its corresponding EDX spectrum. (**a**) 600×; (**b**) 6,000×; (**c**) and (**d**) 10,000×.

**Figure 6:**
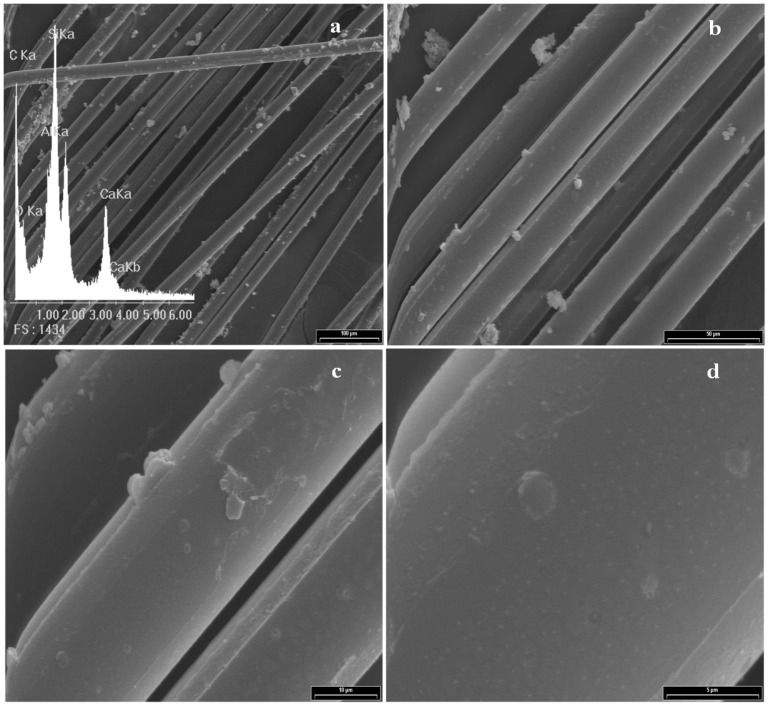
SEM micrographs for sample F1.5% and its corresponding EDX spectrum. (**a**) 200×; (**b**) 600×; (**c**) 2,000× and (**d**) 6,000×.

**Figure 7:**
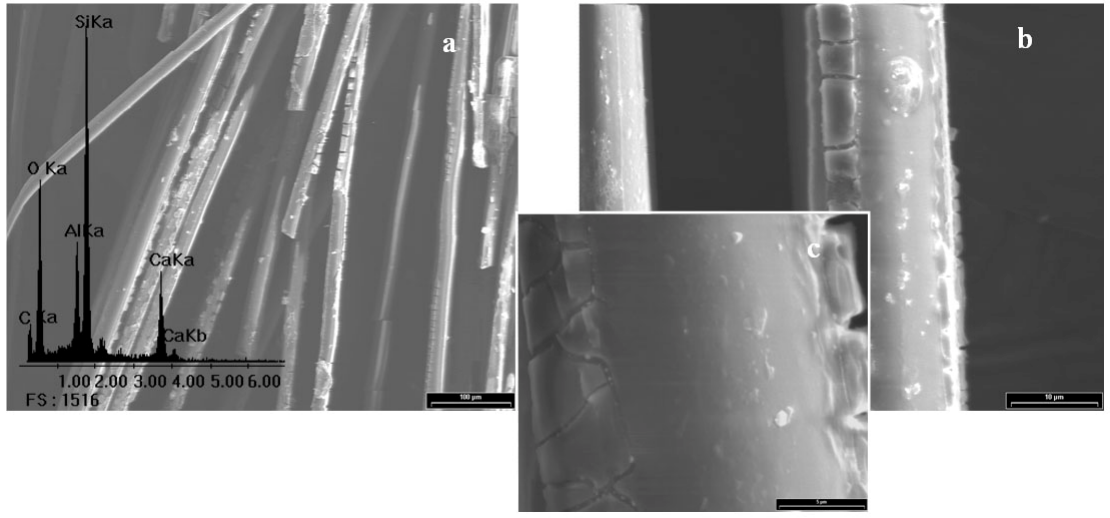
SEM micrographs for sample F2% and its corresponding EDX spectrum. (**a**) 200×, (**b**) 2,000× and (**c**) 6,000×.

Figure 3 shows the scanning electron micrograph of GFs without any treatment and its corresponding EDX spectrum, which is used as standard. Peaks of Si, O, Al and Ca for the E-glass fiber chemical composition can be seen. Note that the peak of C is undetectable, so it is not possible to establish an initial C/Si ratio. A complete morphological study was performed on all reaction products in order to optimize the alcohol concentration that maximizes the PP-glass adhesion. Figure 4, Figure 5, Figure 6 and Figure 7 show the fiber surfaces after *in-situ* polymerization for all hydroxy-α-olefin concentrations. The EDX spectra are also included to verify the nature of the species attached to the fibers; and to allow a rough estimation of their relative amounts. In all cases, the polymers attached to the fibers appear as white particles in the SEM micrographs. From the EDX spectra, it is clear that the C peak increases notably after polymerization.

For all *in-situ* polymerizations, the EDX spectra show large C peaks and obviously O, Al, Si and Ca. The C peak corresponds to PP adhered to the glass surface. Note that in some cases, when the amount of C is very large (F1% in Figure 5) the typical peaks from the fiber (Si, Al, O) are relatively reduced, as expected. In low magnification, the micrographs show that the degree of coverage for different concentrations of hydroxy-α-olefin used is different.

In extreme conditions (F0.5% and F2%) the coverage is not homogeneous, presenting zones with larger polymer agglomerates. At high magnifications, it is evident that the polymer onto fibers presents different morphologies. These crystalline forms are typical of the crystallization during polymerization of polyolefins, particularly PP. Wunderlich shows similar results for PP, where the difference between the spherical macroscopic crystalline forms attached to the polymer is first generated and then crystallized, nucleated by a particle of the catalyst. The smaller rod-shaped particles are those where the crystallization occurs as the polymer is formed [42,43].

Taking into account that the objective is the improvement of adhesion, a homogeneous polymer layer on the fibers is needed. F1% and F1.5% samples show the highest degree of coverage while F0.5% and F2% samples have agglomerated polymer. The greater the amount of hydroxy-α-olefin, the greater the number of anchor points and the lower the PP chain length. Aaltonen *et al.* [44,45], report that an increase in the concentration of hydroxy-α-olefin reduces the rate of consumption of propylene when other polymerization conditions are kept constant..

From these results it appears that the length of the PP chains decreases when the concentration of hydroxy-α-olefin increases. These observations also agree with the EDX results. Comparing the EDX spectra, it is clear that in all polymerizations the C peak appears, but its relative intensity is much greater for conditions F1% and F1.5% that for F0.5% and F2% samples.

**Figure 8:**
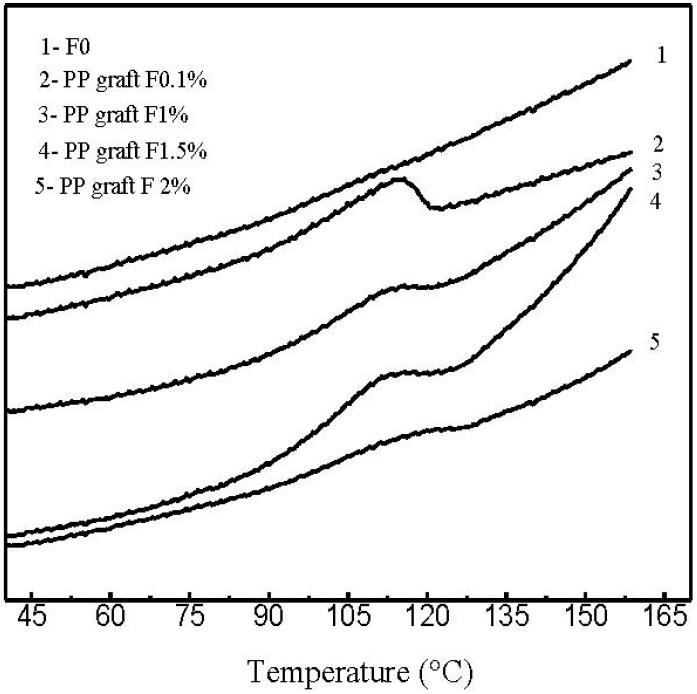
Differential Scanning Calorimetry (DSC) thermograms of the polymer grafted onto the GFs with different hydroxy-α-olefin concentrations.

**Figure 9:**
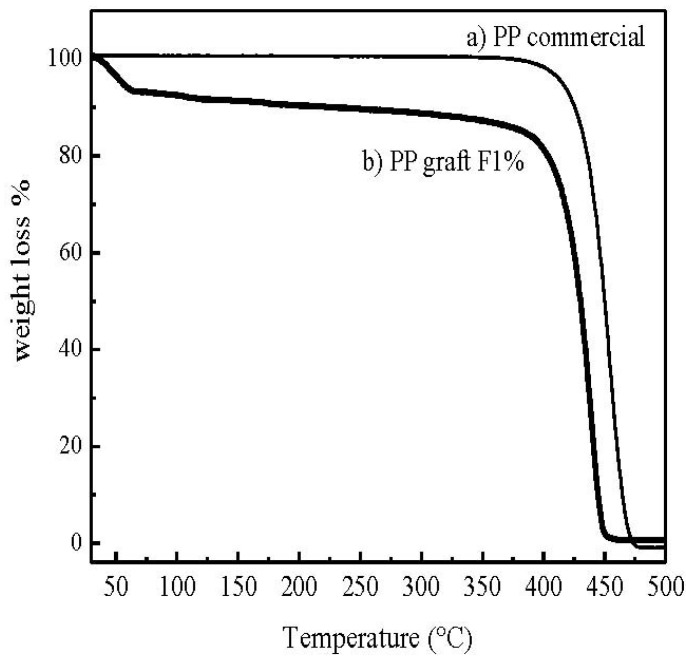
Thermogravimetrical analysis (TGA) thermograms for the sample (**a**) PP commercial and (**b**) PP graft MF1%.

Thermal characterization of the polymer grafted onto fibers was performed by Differential Scanning Calorimetry (DSC) and Thermogravimetrical analysis (TGA). Figure 8 shows the DSC thermograms of the PP grafted onto fibers with different percentages of hydroxy-α-olefin and compared with untreated fibers. Low melting temperatures (110–122 °C) in PP copolymerized onto fibers is typical of γ-PP metallocene polymerization. TGA gives the evidence that molecules grafted onto the fibers at the optimal condition are PP ones. The thermograms obtained allow the evaluation of the mass changes that occur at different temperatures, indicating the transformations taking place at all times. Figure 9 shows the weight loss as a function of temperature. Degradation of PP can be observed at 425 °C whereas commercial PP degrades at approximately 450 °C.

**Figure 10:**
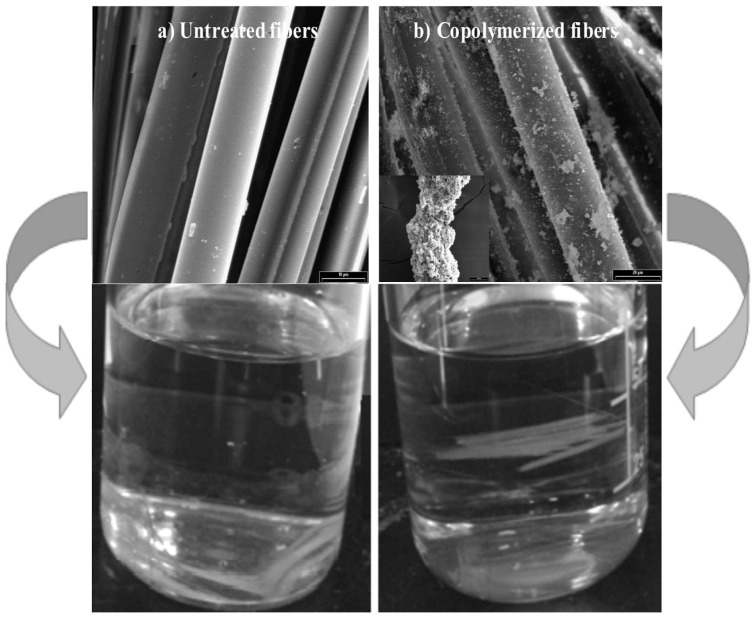
SEM micrograph (2,000×) and photograph of hydrophobicity test of: (**a**) untreated fibers and (**b**) copolymerized fibers.

The polymer fiber coverage after *in-situ* polymerization and previous to composite preparation was qualitatively determined by a hydrophilic/hidrophobic test. The results are interpreted in terms of surface energy (or activity) change due to the attached material to GF. This technique includes the immersion of fibers in a mixture of two immiscible liquids with different polarity and density. A mixture of 50% v/v hexane/water was used in these tests. The densities at 20 °C were: hexane 0.675 g/cm^3^ and water 1 g/cm^3^. The GF surface behaves, initially, hydrophilicly since its superficial hydroxyl groups tend to form hydrogen bonds with water. However, such hydrophilicity should disappear as a consequence of the surface copolymerization and the resulting “PP coverage”. The different morphology exhibited by the fiber surface before and after copolymerization with 1% hydroxy-α-olefin is shown in Figure 10. The complete PP coverage onto the GF is apparent from the comparison of Figure 10a, b. This is consistent with possible surface activity variations that occurred with the polymerization onto the fibers. Untreated GF, which have surface hydroxy groups [1], are hydrophilic, hence they sunk in water, as their density is 2.5 times greater. However, propylene copolymerized fibers remained at the water-hexane interphase. Since the “coating” is non-polar, the fibers became more compatible with the organic phase. Then, as Figure 10 clearly shows, the “attraction forces” of the organic phase are greater than the gravity effect, showing that the GF surface energy radically changed. This simple test gives a qualitative but strong indication of the surface change that occurred to the fibers with the copolymerization treatment.

## 5. Adhesion Improvement Assessing

### 5.1. Theoretical Background

Fragmentation tests were used to assess whether the polymerization technique increments the interfacial adhesion. Interfacial shear strength (ISS) is the result of static equilibrium between the tension force acting on the fiber and the shear force transferred through the interface of the matrix to the fiber [46,47]. According to the Kelly-Tyson approach [48], this value is defined as

(1) $$ISS=\frac{{\bar{\sigma }}_{fb(Lc)}d}{2Lc}$$

where ${\bar{\sigma }}_{fb(Lc)}$ is the mean fiber strength at the critical length, *Lc*, and* d* is the diameter of the fiber.

The value of the critical fragment length can be determined from the average length at saturation (Ls), assuming that the distribution of the fiber fragment lengths at the end of the rupture process is quasi-symmetrical:

(2) $$Lc=\frac{4}{3}Ls$$

It is clear, from Equation (1) that the calculation of ISS implies that it is known how the fiber strength depends on the fiber length. In the present work, this relation was experimentally assessed by carrying out single filament tensile tests following ASTM 3379 standard [49]. These tests were conducted at a given gauge length and the data were fitted by a two-parameter Weibull distribution. According to the ‘weakest link’ approximation, it is assumed that a fiber of a length *L* is formed by *N* independent links of arbitrary unit lengths *L_0_*, each link failing or surviving at a given stress level, independent of its neighbors. The strength distribution of each link is also described by a simple Weibull distribution, characterized by identical shape parameters. Then, the Weibull cumulative distribution function F(σ), and the corresponding average strength, ${\bar{\sigma }}_{fb(Lc)}$, respectively, are given by:

(3) $$F(\sigma )=1-\exp\left[-L{\left(\frac{\sigma }{\sigma_{0}}\right)}^{m}\right]$$

(4) $${\bar{\sigma }}_{fb}\;(L)\;=\sigma_{0}{\left(\frac{L}{L_{0}}\right)}^{-\frac{1}{m}}\Gamma \left(1+\frac{1}{m}\right)$$

where Γ is the Gamma function. The scale (σ_0_) and shape (*m*) parameters of the Weibull distribution were estimated from strength data determined at one single gauge length by fitting the distribution of failure probability. The fiber tensile strength at any gauge length needed for the calculation of ISS was finally calculated on the basis of Equation 4.

**Figure 11:**
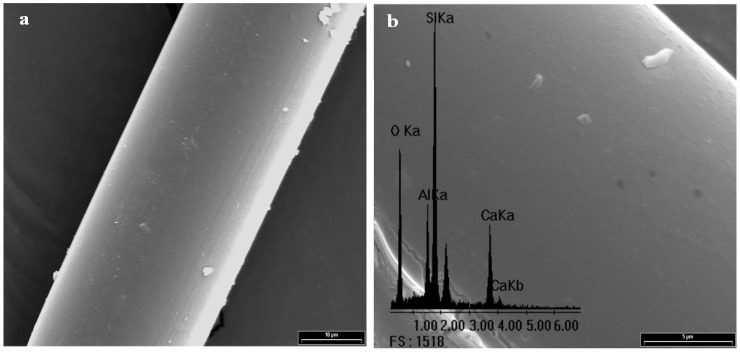
SEM micrographs of the sample MAO-F and its corresponding EDX spectrum. (**a**) 2,000× and (**b**) 6,000×.

The mechanical strength of the fibers was measured on the starting sample; untreated and only MAO treated fibers. Notably, the latter study is to analyze whether the attack with MAO produces any change in the fibers’ resistance. These changes may stem from a possible smoothing, including micro-cracks, surface hardening *etc.* Importantly, a preliminary observation with SEM/EDX of the fibers only attacked with MAO was done and the results are shown in Figure 11.

In principle, it can be seen that a very smooth surface with a metallic brightness due to the aluminum oxide layer is formed. Its appearance in general does not differ from that of the starting GFs. Microcracks are not observed, however the final conclusion will only be possible by measuring the mechanical properties of the fibers. In turn, the EDX spectrum shows that treatment with MAO does not introduce major changes in the surface composition of fibers with respect to the starting GFs. No C peak can be seen and the Al/Si is maintained. Only an increase of peak O is observed, which is expectable due to the formation of the oxide. The fibers were characterized mechanically using tensile tests according to ASTM 1557. The fiber diameter was measured under an optical microscope. Average mechanical properties of fibers and their diameters are summarized in Table 2 where it can be seen that, in principle, the fibers remain unchanged, because the changes in mechanical properties are within the experimental error of measurements.

**Table 2:** Average mechanical properties of fibers and their diameters.

Fiber	Diameter [μm]	Young Modulus E [GPa]	Strength at break (σ_b_) [MPa]	Elongation at break ε [%]
GF	26.8 ± 1.8	46.11 ± 5.7	1,688 ± 206	3.02 ± 0.29
MAO-GF	25.1 ± 1.0	48.71 ± 4.2	1,516 ± 163	2.94 ± 0.42

**Table 3:** Weibull parameters for a reference length L0 = 25 mm [50].

Fiber	Scale parameter *σ_0_* [MPa]	Shape parameter *m*	χ^2^	R
MF0	1,556	2.36	0.122	0.976
MAO-GF	1,361	2.92	0.029	0.986

**Figure 12:**
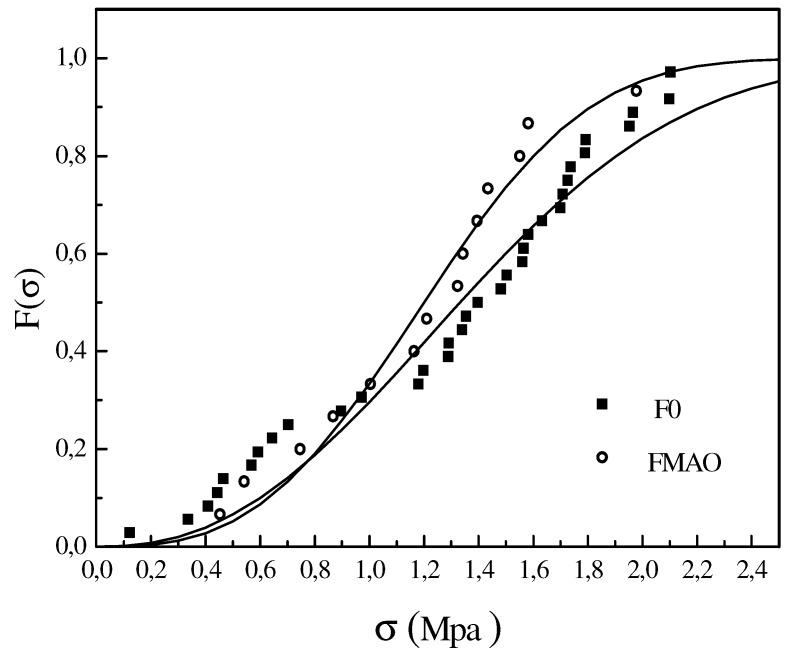
Weibull plot of untreated and MAO-treated fibers [50].

Tensile tests on both untreated and MAO treated single GFs were performed to determine their strength. The strength data were statistically treated according to the Weibull distribution. The results are summarized in Figure 12. It is worth to note that the MAO treatment induced only a slight decrease in the fiber tensile strength as documented by the small reduction in the Weibull scale parameter, r_0_, reported in Table 3. At the same time, a slight increase of the Weibull shape parameter was found, m, wich accounts for a somewhat sharper distribution of strength values of the treated fibers around an average value. These results are in agreement with the morphological observations reported above and provide further evidence that MAO treatment does actually induce very limited fiber damage. The effects of this small reduction of the fiber strength on the interfacial adhesion have been taken into account by considering the actual values of the fiber strength at the critical length [50].

### 5.2. Interfacial Shear Strength Evaluation

**Figure 13:**
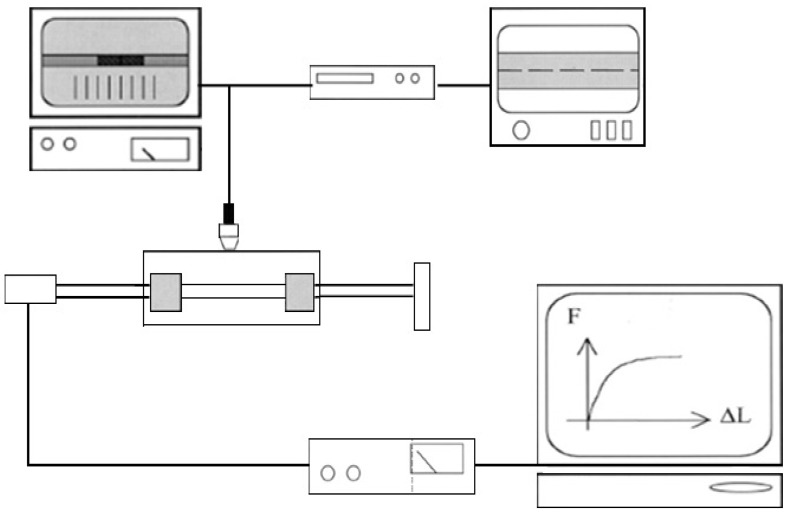
Single fiber fragmentation test set up.

The interfacial adhesion was determined by single fiber fragmentation tests, carried out on model composites consisting of a single GF embedded in a PP matrix. Microcomposite samples were prepared by compression molding. Fibers were carefully aligned between two PP films and in turn sandwiched between two stainless steel sheets. Microcomposites were prepared using untreated and treated fibers with 0.5%, 1.0%, 1.5% and 2.0% of hydroxy-α-olefin, and they were named *PP/F (% hydroxy-α-olefin)/PP.* Single fiber fragmentation tests were performed on model composites consisting of a single fiber embedded in a polymeric matrix. It was measured at room temperature by using a custom-made apparatus as shown in Figure 13 [50,51]. The mean fiber length, Ls, was measured by means of an image analyzer system, as proposed by Ohsawa *et al.* [52].

**Figure 14:**
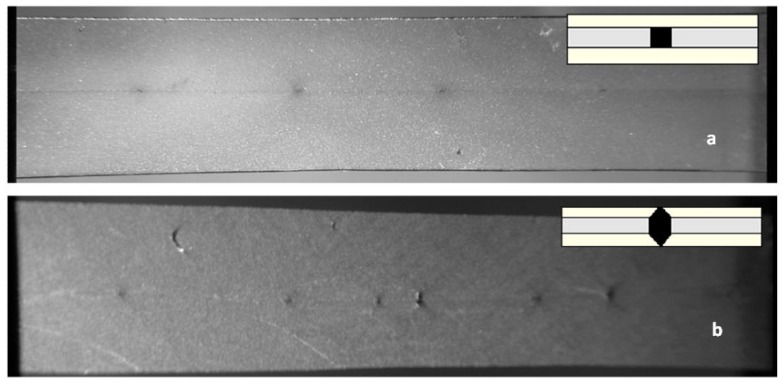
Optical microphotographs (4×) from fragmented fibers in microcomposites (**a**) PP/F0/PP and (**b**) PP/F1%/PP.

An example of the fiber failure appearance is reported in Figure 14 for PP/F0/PP and PP/F1.0/PP. For the untreated fibers (Figure 14a), the interfacial debonding appears to be the dominant failure mechanism, thus indicating low-adhesion conditions. On the other hand, a different failure behavior is observed for microcomposites with fibers previously treated with 1.0% of hydroxy-α-olefin. Here, a radial matrix crack or mixed radial-conical cracks occur (Figure 14b). This behavior is an indication of a somewhat better fiber matrix-adhesion, as reported by Pegoretti *et al.* [51,53]. From Figure 14 also clearly shows that the number of fiber fragments increases with the interfacial adhesion, resulting in a shorter critical length, as summarized in Table 4.

**Table 4:** Average fiber critical length, Lc and Interfacial shear strength (ISS) for all composites [50].

Sample	Average fiber critical length, *Lc* [mm]	Interfacial shear strength *ISS* [MPa]
PP/F0/PP	8.43 ± 1.85	3.5 ± 0.8
**PP/F1%/PP**	**4.10 ± 0.64**	**7.4 ± 1.1**
PP/F1.5%/PP	4.94 ± 1.19	5.8 ± 1.4
PP/F2%/PP	4.49 ± 0.34	6.5 ± 0.5

**Figure 15:**
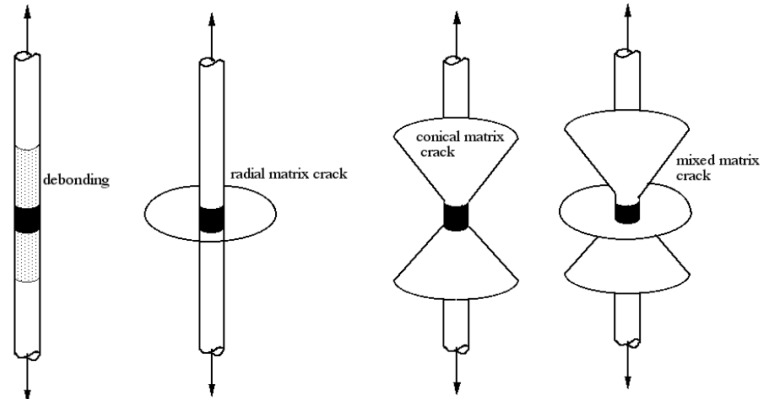
Fracture model proposed for simple fragmentation test [53].

Figure 14 shows the appearance of microcomposites after the fragmentation test for samples prepared using untreated fibers and treated fibers in optimum condition (1% hydroxy-α-olefin). These photographs, taken with optical microscopy equipment, indicate that in the untreated fibers, the segments are long, the remaining space between segments is shaped “cylindrically” and the crack appears to have spread into the matrix (Figure 14a). Moreover, in the case of fiber microcomposites copolymers, the segments are significantly shorter and the space segment-segment has a different shape, similar to that of a double truncated cone whose vertex is set off from the ends of the fiber segments. Additionally, the crack is spread widely in the matrix (Figure 14b). In the literature three possible failure modes from fragmentation tests are reported. Debonding failure, where the crack propagates parallel to the interphase, is the typical case of a very poor adhesion with low interfacial resistance. If the resistance is higher, the crack can propagate to the array before the fiber falls away. This behavior manifests itself in a disk perpendicular to the rupture of the fiber, and interfacial resistance is typical of socks. The third case, when adherence is very good, a cone-type fracture is observed with a vertex at both ends of the broken fibers. Importantly, the behavior intermediate between those described presents as a combination of these patterns of fracture [53]. Figure 15 shows these models schematically. Clearly, the results obtained (shown in Figure 14) describe two extreme cases. No adherence seems to be the dominant failure mechanism in the case of PP/F0/PP microcomposites showing poor interfacial adhesion, on the contrary, the samples that have broken polymerized matrix-radial cone type fractures from the interface of the fiber indicate a marked improvement in adhesion.

In the same table, the interfacial shear strength, calculated in accordance to Equation (1), is also reported. The adhesion markedly increases when fibers with *in-situ* polymerization are considered. For microcomposites obtained with polymerized fibers using 1% of hydroxy-α-olefin, an ISS value more than two times higher than that of the untreated fibers is observed. On the other hand, it appears that the improvement of interfacial adhesion decreases as the amount of hydroxy-α-olefin used in polymerization decreases. This behavior could be explained in terms of a competition between the length of the growing PP chains and the number of anchorage points generated on the fiber surface. In fact, the higher the amount of hydroxy-α-olefin, the higher the number of anchorage points and the lower the PP chain length. The anchorage is formed by the reaction between the hydroxyl groups of the hydroxy-α-olefin and the aluminoxane from MAO, which is bonded to the glass surface [50]. Then, as the hydroxy-α-olefin concentration increases, the number of anchorage points also increases. However, the presence of hydroxy-α-olefin (a polar compound) results in a decrease of the catalyst’s activity. Aaltonen *et al.* [44,45] showed that the synthesis of hydroxyl group containing polyolefins with metallocene/methylaluminoxane catalysts displayed a polymerization rate with decay type kinetics. In particular, these authors reported that an increase of hydroxy-α-olefin concentration reduced the consumption rate of propylene when the other polymerization conditions were kept constant. Hence, it appears from their results that the PP chain length between anchorage points decreases with the hydroxy-α-olefin concentration. Since interfacial adhesion is developed mostly by PP molecular entanglements, the incidence of changes in chain lengths should be more important than the number of anchorage points.

### 5.3. Microcomposites Interphase Characterization

**Figure 16:**
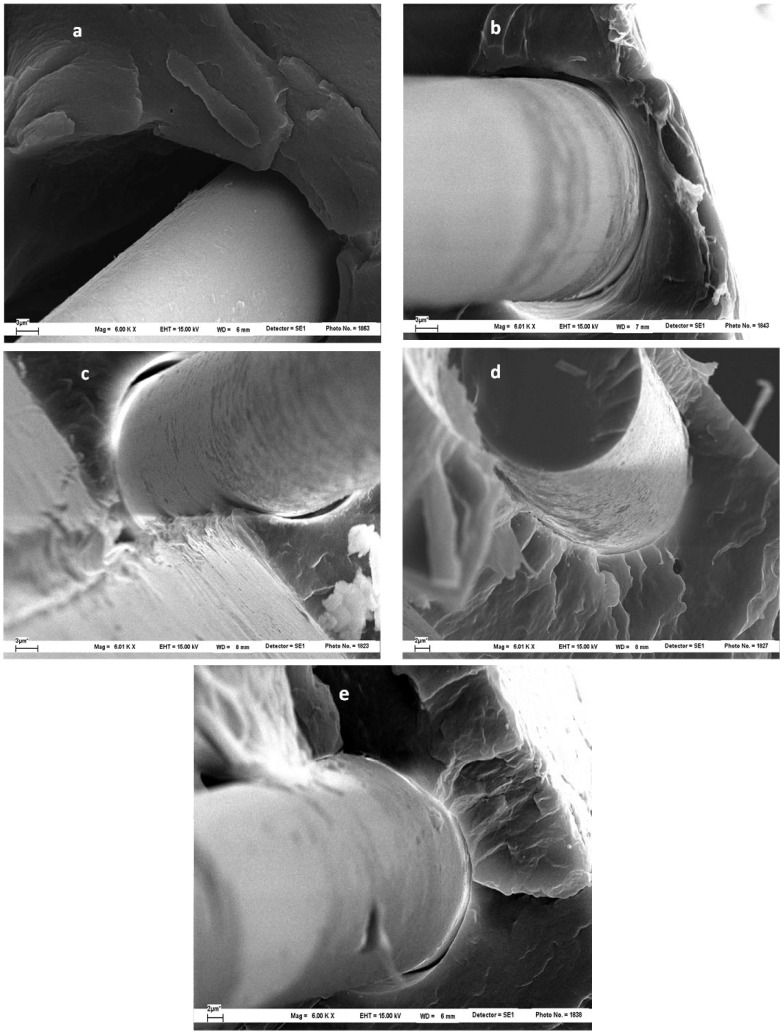
SEM microphotographs (6,000×) from cryogenic fracture surface of different microcomposites: (**a**) PP/F0/PP; (**b**) PP/F0.5%/PP; (**c**) PP/F1%/PP; (**d**) PP/F1.5%/PP; (**e**) PP/F2%/PP [51].

**Figure 17:**
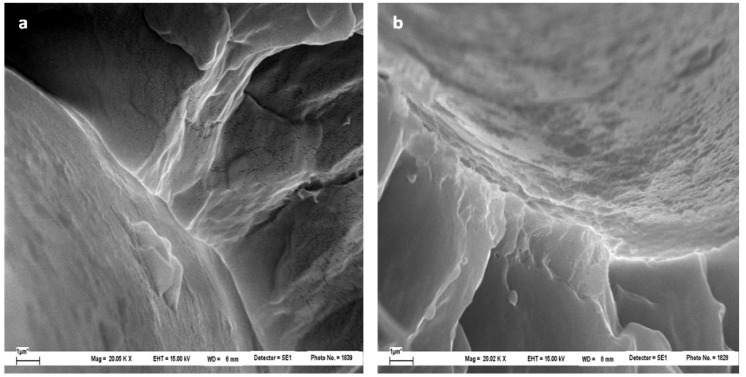
SEM micrographs (20,000×) of the composites (**a**) PP/F1%/PP and (**b**) PP/F1.5%/PP [51].

After the fragmentation test, the morphology of cryogenically obtained fracture surfaces of microcomposites was analyzed by SEM. Figure 16 shows the appearance of both untreated and treated samples with different concentrations of hydroxy-α-olefin, as observed at 6,000× magnifications. For the sample PP/F0/PP, the neat fiber pullout and the absence of polymer attached to the fiber surface clearly indicate a very limited adhesion between PP and the fiber (Figure 16a). On the other hand, the fracture surfaces of microcomposites made with *in situ* polymerized fibers show features indicating a better fiber-matrix adhesion. In fact, as documented in Figures 16b–e, PP partially adheres to the fiber surface. Figure 17, at higher magnification, also shows that PP is adhered to the the fiber surface of F1.0 and F1.5 samples. The increment in adhesion is also demonstrated by the radial fracture cracks emanating from the fiber interface (Figure 16d, e and Figure 17b). This latter feature indicates that the PP-glass interface strength is more stable than the PP–PP entanglements and the fracture occurs in the PP region around the fibers, not at the interface.

**Figure 18:**
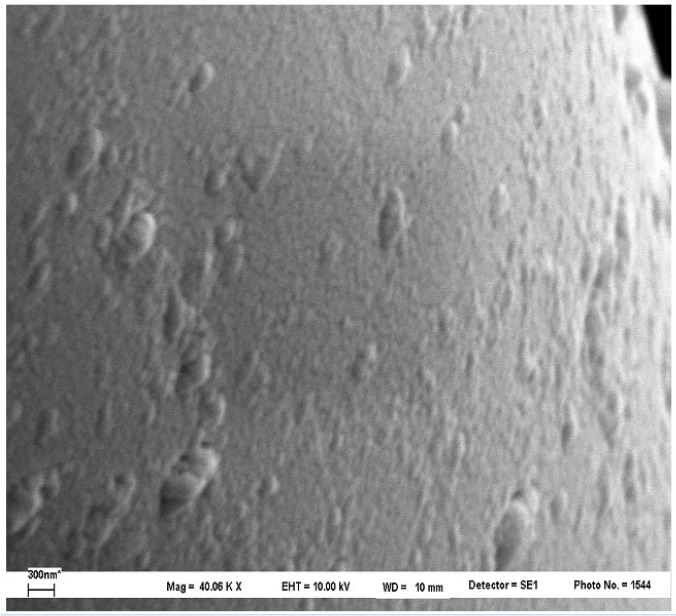
SEM micrographs (40,000×) of fiber surface after cryogenic fracture of PP/F1%/PP [51].

Details of the fiber-matrix “interphase” for the two best conditions (1% and 1.5%) are shown in Figure 17. In these it is clear that the fiber-matrix bond is retained after the fracture (micro-void). Much of the polymer bonding remains and is evenly distributed on the fiber surface. Major approaches in other areas of the fiber surface after fracture (Figure 18) confirm the earlier observations. The polymer is evenly distributed on the fiber surface and is seen as a bump smaller than 200 nanometers. This is consistent with the polymer attaching to the surface and then splitting at room temperature and returning to its “curled up” structure.

These observations support the hypothesis that the fracture in the fiber microcomposites copolymers preferably occurs in the polymer mass surrounding the fiber, with the entanglement breaking off rather than splitting between the glass and the polymer, confirming that the technical compatibility proposal greatly improves adhesion.

## 6. Mechanical Properties of PP/GF/PP Composites

Tensile mechanical behavior of laminated PP/GF/PP was evaluated using unidirectional arrays. The mechanical properties obtained were interpreted according to the adhesion results and phenomenological evidence.

The tensile behavior of composite materials is difficult to predict, as the fracture mechanics are so complex. Is known that the composite shows a marked anisotropy, that is to say, its properties vary significantly when measured in different directions. This usually arises because the harder constituent is in a fibrous form with the fiber axes preferentially aligned in particular directions [3]. Hence, samples were prepared with uni-axially oriented continuous fibers, where the rule of mixtures can be used to predict modulus and strength. According to this rule, the mechanical strength for continuous, uni-axially oriented fiber composites is just based on the properties of each component and the relative concentration and orientation of the fibers. These approaches assume that the charge transfer is complete and the variations in adhesion between the fiber and the matrix are not taken into account. For this reason, they are, in principle, more suitable to predict the modulus value than the mechanical strength. The elastic modulus is measured as the slope of the stress-strain curve at low deformation, as the charge transfer is less critical in this property determination. Very low deformation materials react to an atomic-molecular level separately. However, the mechanical strength is the maximum tension that the material can resist, and it usually occurs at higher deformation, where charge transfer is a decisive factor, and consequently, adhesion influences its value. It is remarkable that the mechanical strength values, calculated from rule of mixture, assume full adhesion and thus overestimate the actual value. This problem is most evident in the case of transverse mechanical resistance, where the matrix “strip” of the fibers goes through the interface. For this reason the equation arises only as an approximation. Tensile tests were carried out on PP, GF and composite samples. Table 5 summarizes the mechanical properties of the PP matrix and the fibers used in the composites. Note that these values are used as parameters, because they were measured under similar conditions with samples of equal size.

**Table 5:** Mechanical properties of PP and GFs.

Property	PP	GF
E [MPa]	685 ± 69.6	47,600 ± 2,015
σ_u_ [MPa]	25.7 ± 1.7	234 ± 31.3
ε_y_ [%]	10.0 ± 1.8	----
ε_b_ [%]	412 ± 92	2.6 ± 0.9

The fiber concentration of the tensile specimens was measured by weight difference before and after washing them. The achieved weight and volumetric fractions are summarized in Table 6 [41].

**Table 6:** Average concentration of fiber composites.

Fraction [%]	PP/GF/PP	PP/MAO-GF/PP	PP/COP-GF/PP
Volume	53.8 ± 5.9	50.8 ± 4.8	46.6 ± 5.3
Weight	76.4 ± 4.35	74.2 ± 3.66	70.8 ± 4.58

Continuous parallel fiber PP/GF composites, containing about 1800 aligned GF, were prepared by compression molding in a hydraulic press. The fibers were carefully aligned and compounded by sandwiching them within two PP sheets. Before compounding, the GF received different surface conditioning: no pretreatment, *in situ* polymerization and just MAO treated surface. These composites were named PP/GF/PP, PP/COP-GF/PP and PP/MAO-GF/PP, respectively. Rectangular specimens for tensile tests were obtained by careful cutting with a sharp blade in order to have net edges and minimize errors during experimental tensile measurements.

**Figure 19:**
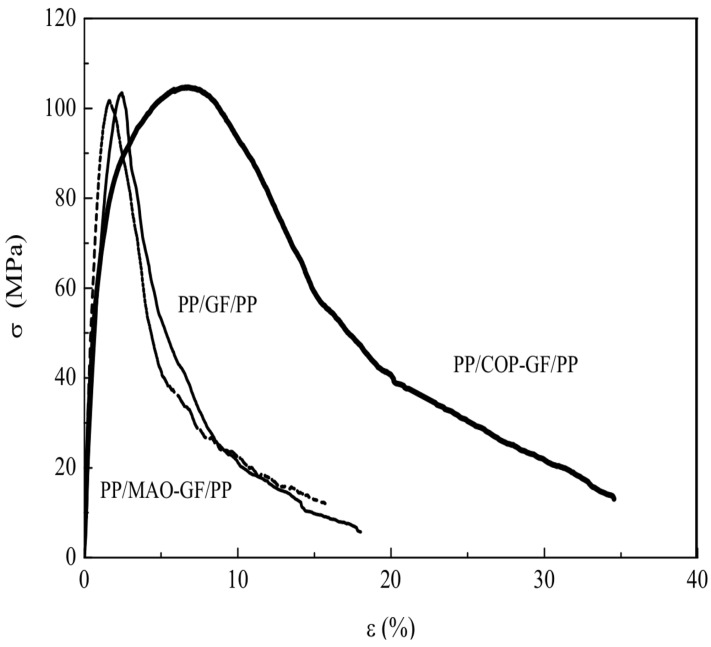
Stress-strain curves of PP/GF/PP composites.

The fracture shape, as well as the morphological features of fiber surface and microcomposites subjected to mechanical tests were studied by SEM. Figure 19 shows tensile stress-strain curves for all the composites tested, PP/GF/PP, PP/MAO-GF/PP and PP/COP-GF/PP. The corresponding properties are summarized in Table 7.

**Table 7:** Mechanical properties for the composites PP/GF/PP.

Property	PP/GF/PP	PP/MAO-GF/PP	PP/COP-GF/PP
E [MPa]	8,125 ± 1,060	8,491±1,029	8,852 ± 1,017
σ_u_ [MPa]	101.6 ± 14.1	103.4 ± 12.3	104.7 ± 11.1
ε_ y _[%]	2.5 ± 0.8	1.6 ± 0.4	6.8 ± 0.9
ε_ b _[%]	14.1 ± 8.3	16.56 ± 6.4	35 ± 7.2
Toughness [J]	12,654 ± 1,100	12,054 ± 512	38,579 ± 1,600

The possible occurrence of fiber damage caused by the MAO attack was studied in a previous study [50], in which tensile stress-strain tests on both untreated and MAO treated single GFs were done. The strength data, statistically treated according to the Weibull distribution, showed that MAO induced only a slight decrease in the fiber tensile strength, indicating that a very small fiber damage could occur in the presence of MAO. This trend is also confirmed for composite samples, since only a small variation in strength and elongation properties was observed for composites having either untreated and MAO treated fibers (Figure 19).

From the strain-dependent tensile behavior, it is observed that the mechanical strength of PP/GF/PP and PP/MAO-GF/PP composites is reached at 1 to 3% deformation; while for PP/COP-GF/PP, the deformation is close to 7%, *i.e*., three times larger. This is related to the composite fracture mechanism. In the first two, the whole composite fracture occurs by fiber breakage, at ε < 3%, without any matrix contribution to resistance due to the low matrix-fiber adhesion. On the other hand, for copolymerized fiber composites, the fibers start breaking at about 2% strain (Figure 19), according to the main break strain of GF. However, the strain (at which strength is achieved) increases up to 7%, and can be attributed to a greater matrix/fiber adhesion. As was demonstrated above, the copolymerized fiber breaks into much smaller segments than samples containing untreated fibers. Then, although the first fracture occurs at the same strain for both treated and untreated fibers, the treated ones keep breaking as the deformation increases over this strain. This is due to the more effective matrix/fiber load transfer that requires greater energy consumption to fracture. In composites with polymerized fibers, GF/PP interface essentially resists by chemical anchorage. Once the fibers fracture, composites do not break immediately. This fact can be assigned to the greater stability of the glass-PP interface as compared to PP-PP entanglement resistance.

**Figure 20:**
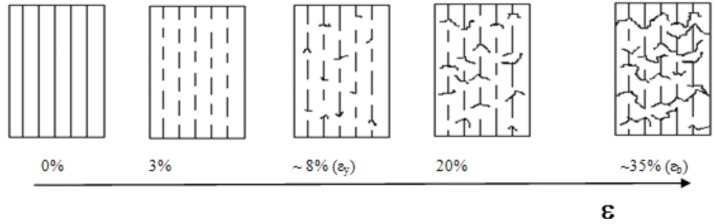
Scheme proposed to explain the stress-strain behavior of PP/COP-GF/PP composites.

The composite initially contains continuous and aligned fibers, but once the first fiber fracture occurs, it gradually converts into a short fiber composite. Here the fiber fragments remain aligned with the load direction and well attached to the matrix. As the strain increases, each fiber fragment bears a growing stress up to its rupture limit. Significantly, the rate at which the load is transferred to the fibers decreases as voids begin to form and grow at the fiber tips. Figure 20 outlines this mechanism.

It is clear that fibers always break at their fracture limit, however the mechanism for crack propagation, and consequently the composite mechanical strength, will be quite dependent on the fiber/matrix adhesion. When adhesion is low (composites with untreated fiber) the crack propagates mainly by fiber debonding. It results in fewer and relatively longer fiber segments. Instead, when polymerized (treated) fibers are used, cracks spread radially, preventing the fiber pullout. In this case, the propagating crack finds a greater amount of bonded fiber fragments in its way; so more energy is needed to break (or evade) them and allow the crack to grow. Therefore, when a large amount of short and well-adhered fiber segments are present, a considerable barrier to crack propagation is set, essentially by increments of the fissure path tortuosity. The proposed mechanism justifies the increment in elongation at break (more than twice for polymerized fiber samples as compared to PP/GF/PP and PP/MAO-GF/PP), as is shown in Figure 20. Also, the toughness, calculated as the composite energy to break from the area under the stress-deformation curve, resulted three times greater for the treated fiber samples (see Table 7). Furthermore, the elongation at yield (related to the energy required to generate cracks) indicated that PP/COP-GF/PP needed 5% more elongation than composites with non-copolymerized fibers to initiate cracks.

This evidence further validates the proposed mechanism. Other authors found similar behaviors, but attributed it to the ability of coupling agents to change the matrix nature, which could act as impediment of crack propagation. These authors did not report an increase in the strain at which the maximum stress occurs, but proposed just an increase of matrix ductility by the coupling agent use as the main cause of improvement [54,55,56].

**Figure 21:**
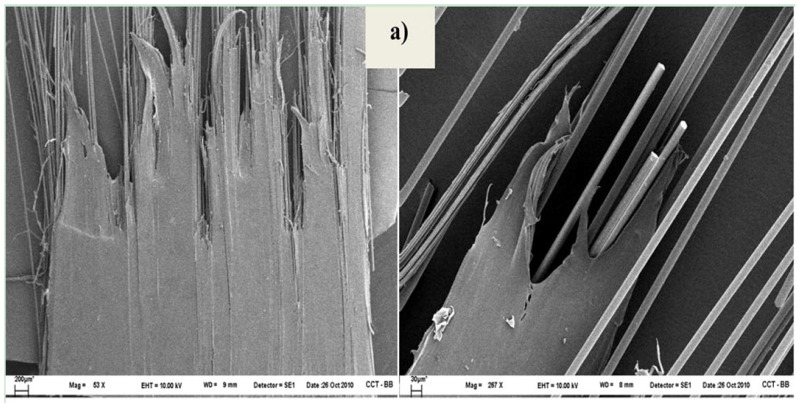

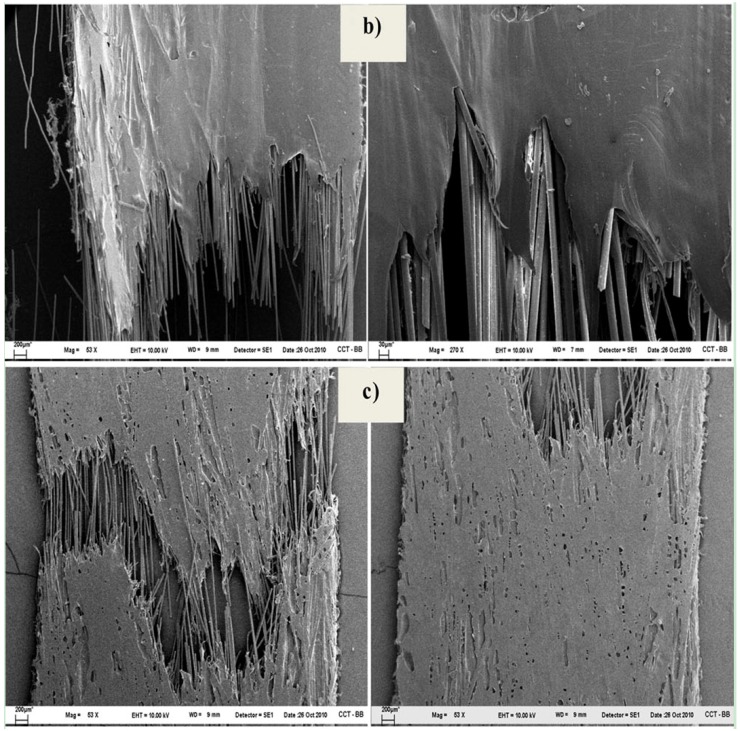
SEM micrograph of composites after tensile break with different magnifications (**a**) PP/GF/PP at 53× and 270×; (**b**) PP/MAO-GF/PP at 53× and 270×, and (**c**) PP/COP-GF/PP at 53×.

**Figure 22:**
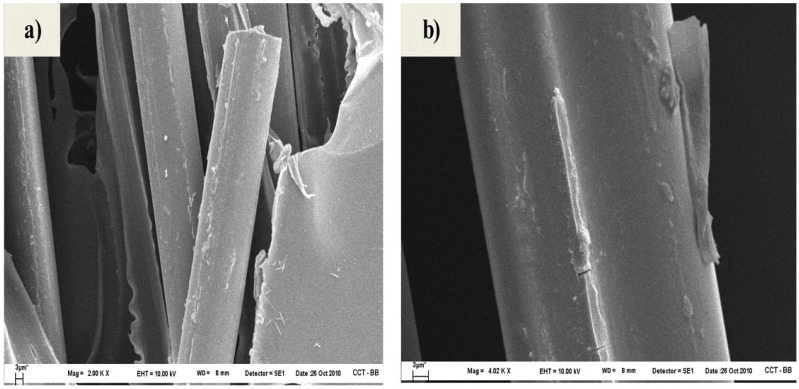
SEM micrographs of PP/COP-GF/PP at: (**a**) 2,000× and (**b**) 4,000×.

In order to corroborate the proposed fracture mechanism, and the adhesion enhancement, SEM characterization of specimens after tensile tests was carried out. Figure 21 shows the fractured sample surfaces of PP/GF/PP, PP/MAO-GF/PP and PP/COP-GF/PP. It is clear from Figure 21a, b that untreated and MAO treated composites breaks mainly by fiber debonding, hence PP remains unchanged. On the other hand, in PP/COP-GF/PP samples, the PP presents a lot of holes evenly distributed throughout the sample, indicating that fibers pull the matrix before composite fracture (Figure 21c). This is consistent with the greater resistance of chemical anchorage relative to PP molecular entanglements. Moreover, the less regular overall fracture is consistent with a greater resistance to breakage. Also, PP patches remain adhered to the fiber surface after the composite fracture (see Figure 22). The observed behavior agrees with the above-proposed model. Here the interphase presents a micro-damage mode of debonding accompanied by matrix cracks and promotes a stronger adhesion between matrix and fibers. The result is better resistance to development of damage and thus higher normal tensile stress. The interface debonding can reduce the effect of the matrix crack. The breakage (instead of debonding) of several small fiber bundles triggers larger matrix cracks, causing their propagation along the longitudinal direction. Table 7 also shows the tensile modulus from non-polymerized and polymerized samples. As it is expected, no appreciable variation is observed since this is a zero strain property and neither fiber nor matrix modulus changed after treatment.

From these results it is clear that the *in-situ* polymerization technique improves the compatibility between phases allowing for more ductile composite materials without affecting their rigidity.

## 7. Conclusions

The technique of *in-situ* metallocenic polymerization of propylene onto a GF surface resulted in a promising route to increase the adhesion between PP matrix and GF reinforcement since an enhanced compatibility between them can be obtained. The main conclusions of this work are:

- PP can be copolymerized onto GFs by using metallocene catalyst.
- PP chains grow from the glass surface and enhance the adhesion by entanglements with PP matrix in polymer composites.
- The amount of PP polymerized onto the fiber and the interfacial adhesion seems to have optimal conditions regarding either the PP coverage or the interfacial shear strength measured.
- The interfacial shear strength of PP/GF composites, measured by the fragmentation test, is considerably improved (up to a factor of 2.1) with respect to the case of untreated fiber-PP composites.
- The radial matrix crack developed during fragmentation tests indicates a good matrix fiber adhesion for all *in situ* polymerized fibers.
- The polymer remains attached to the fiber surface after cryogenic fracture for all the samples subjected to polymerization.
- Uniaxial composites PP-copolymerized GF presents considerably higher ductility and toughness without detriment of strength, with respect to the composites prepared with untreated fibers. These improved properties are assigned only to the increased adhesion caused by molecular entanglements between the PP-grafted GF and PP matrix.
- The MAO pretreatment does not change the PP/GF mechanical properties, since both the adhesion and fiber properties remain unchanged.
- This treatment strongly modifies the composite fracture behavior. The interface fiber debonding is the main mechanism for untreated fiber composites, while PP disentanglement accounts for the breakage of polymerized fiber composites.

The *in-situ* polymerization method is clearly a very good approach to improve the compatibility between GFs and PP. The adhesion enhancement as well as the mechanical properties improvements were demonstrated with the evidences shown above. It also seems to be a promissory method to obtain PP prepregs to increase the performance of PP/GF composites by using mat reinforcements.
